# The Solvation Effect of C=O Group of Cyclic Anhydrides in Solution

**DOI:** 10.3390/ijms24076724

**Published:** 2023-04-04

**Authors:** Ilnaz Rakipov, Aydar Akhmadiyarov, Artem Petrov, Artashes Khachatrian, Boris Solomonov

**Affiliations:** Department of Physical Chemistry, Kazan Federal University, Kremlevskaya 18, Kazan 420008, Russia

**Keywords:** IR spectroscopy, cyclic anhydride, dipeptide, solvation effects, hydrogen bonding, multi-particle complexes

## Abstract

The paper reports the results of investigation intermolecular interactions between alanine and sarcosine anhydride in organic solvents. The absorption frequencies of cyclic dipeptide in solvents were measured by IR spectroscopy. The effect of Van der Waals interactions and the hydrogen bonding of solvents on absorption frequencies of the C=O group of anhydrides was discussed. The spectroscopic parameters for C=O∙∙∙H-O hydrogen bonding complexes of anhydride with methanol in aprotic and proton donor solvent are obtained. In multi-particle complexes of dipeptides with aliphatic alcohols, the hydrogen bond enhancement was between 10 and 16%, which is significantly lower than it is for amide complexes.

## 1. Introduction

Cyclic anhydrides, also known as diketopiperazines (DKPs), are cyclic dipeptides that are extensively used due to their biological activity [1,2]. The variety of diketopiperazines determines their wide spectrum of biological activities, including antibacterial, antifungal, antiviral, and antitumor properties [1,3]. This is possible due to intermolecular interactions. This system is actively used for drug delivery [4]. The special role of these compounds as targets for antitumor drug delivery has been noted [5,6]. The DKP ring confers increased structural rigidity and stability against proteolysis relative to acyclic peptide scaffolds, making DKP attractive for pharmaceutical development [2]. The special properties of small cyclic dipeptides are related to their ability to form networks of intermolecular hydrogen bonds. These aggregates of cyclic dipeptides that form due to hydrogen bonds can lead to the formation of micro- and nanostructures, which are widely used for practically important tasks [4,7].

It should be noted that intermolecular diketopiperazines are studied with a spectrum of experimental and theoretical methods [8,9,10,11]. This article presents the results of the thermodynamics of the transfer of cyclic dipeptides: alanine–alanine (AA) and glycine–glycine (cGG) between gas, aqueous, and crystalline phases [8]. The authors note that studies of intermolecular interactions in crystals of cyclic dipeptides: glycine–glycine (cGG) and alanine–alanine (cAA) allow us to explain the reason for the decrease in enthalpy with an increase in hydrophobicity both during the dissolution of cyclic dipeptides and during protein unfolding [8]. This decrease arises due to the long-range electrostatic interaction between dipeptide molecules in crystals, which is weakened by more hydrophobic side chains [8]. Thermodynamic parameters of the dissolution of cyclic dipeptides in water were studied in this work [10]. The effect of intermolecular interaction strength between amide groups of dipeptides and the amide–hydroxyl group of water on enthalpic stability was revealed, and it is noted that the strength of the amide–amide hydrogen bond is twice as strong as the amide–hydroxyl bond. The authors present the unusual conclusion that the hydroxyl group exhibits weak hydrophobic properties [10]. In this work studied the conformational features of diketopiperazines that made them unable to achieve hydrogen bonding by X-ray crystallography and density functional theory [11]. The conformational state of diketopiperazines in the gas phase and in the crystal was shown to match. This is associated with an insignificant influence of intermolecular interactions on packing in the crystal in the absence of classical N-H∙∙∙O hydrogen bonds.

Thus, it is important to evaluate the proton acceptor and proton donor properties of diketopiperazines in solutions. In this case, studies on associated solvents, including water, are of particular interest. Earlier, our group actively studied the intermolecular interactions of structural fragments of dipeptides and proteins (linear and cyclic amides) [12,13,14,15,16]. The effect of the number of active proton donor centers of linear amides on their proton donor properties in solution was shown [12]. It was noted that the presence of the amide cycle leads to an improvement in the proton donor properties of the amides [13]. The case when amides are solvent agents was considered, and the formation of hydrogen bonds of proton acceptors in amides was evaluated. Cooperativity and reorganization effects between amides and organic molecules in multi-particle complexes were discussed [14,15]. The realization of the interaction of protein structural fragments through N-H∙∙∙O=C hydrogen bonds was of particular interest, since it is these bonds that determine the stability of proteins [17,18,19]. The stability of these systems is determined by the cooperativity of hydrogen bonds [20,21,22]. We studied the cooperative effects of amides with organic molecules by infrared spectroscopy [16]. The effect of solvent properties on the stretching vibrations of the C=O groups of the amides was studied [16]. A method for the evaluation of cooperative effects in multi-particle amide complexes in self-associated solvents was proposed. It was found that the cooperative effects of *N,N*-dimethylformamide (DMFA) with alcohols are larger in magnitude than those of *N*-methylformamide (NMFA) with aliphatic alcohols. It should be noted that cyclic dipeptides form twice the number of hydrogen bonds per molecule in comparison with those formed by linear amides. 

Therefore, the task was to study the solvation effects of small cyclic dipeptides, alanine anhydride, and its substituted derivative, sarcosine anhydride, in solutions by infrared spectroscopy (IR). Comparative analysis of the intermolecular interactions of cyclic dipeptides with previously studied amides was carried out. 

## 2. Results and Discussion

The effect of intermolecular interactions on the absorption frequencies of the C=O groups of alanine anhydride and its substituted analogue, sarcosine anhydride, in aprotic and proton donor solvents was studied. Comparative analysis of the intermolecular interactions of cyclic dipeptides with previously studied amides was carried out.

### 2.1. Solvation Effects of Anhydrides in Inert and Aprotic Solvents

**Figure 1:**
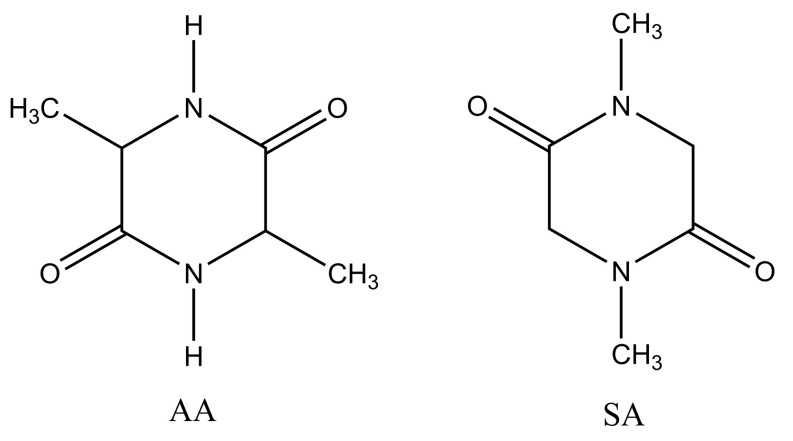
Diketopiperazines studied in this work.

In this section, we studied the absorption frequencies of the C=O groups of dipeptides in aprotic and proton donor solvents. Cyclic anhydrides were chosen as the studied compounds (Figure 1).

**Figure 2:**
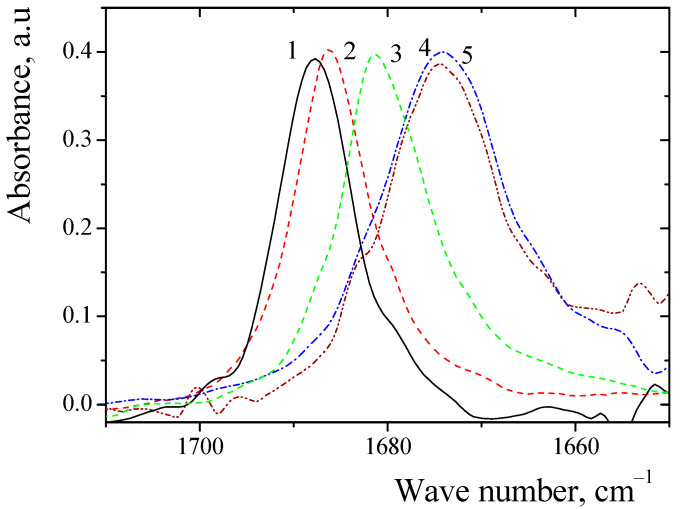
IR spectra of the C=O group of sarcosine anhydride in solvents: 1—tri-ethylamine; 2—diethyl ether; 3—tetrahydrofuran; 4—acetonitrile; 5—nitromethane.

Scans of the spectra indicated their presence in the region from 400 to 4000 cm^−1^. The absorption frequencies of the C=O area was analyzed. The absorption frequencies of the C=O group of sarcosine anhydride in the environment of organic solvents are presented in Figure 2.

Frequencies of the stretching vibrations of C=O groups of alanine anhydride and sarcosine anhydride in solvents are presented in Table 1. The S_VW_ parameter of solvents was taken from [16].

**Figure 3:**
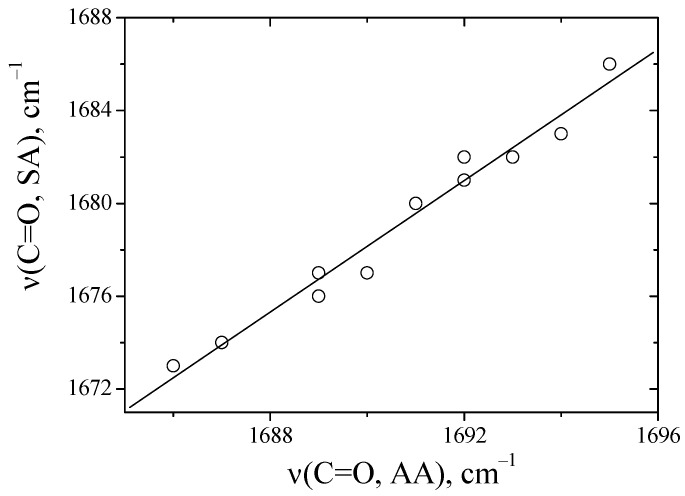
A comparison of the absorption frequencies of C=O groups between alanine (AA) and sarcosine anhydride (SA) in solvents, values correspond to the data in Table 1.

Figure 3 presents a correlation of the absorption frequencies of the C=O groups of anhydrides with each other in solvents.

It was found that there is a linear dependence between N-H and methyl-substituted anhydride in aprotic solvents (Figure 3). Previously, when we compared the absorption frequencies of the C=O groups of *N*-methyl with those of *N,N*-dimethyl-substituted amides, no linear dependence was observed [16]. This was realized through the formation of hydrogen bonds of amides with proton acceptors of the N-H∙∙∙B type. For anhydrides, deviations in the absorption frequencies of C=O groups in the proton acceptor environment are not observed. This is probably related to the conformational features of diketopiperazines [23].

In this work, we used S_VW_ parameters [24,25] to analyze the solvation effects on anhydride absorption frequencies. This parameter allowed us to quantitatively estimate the Van der Waals interactions between the soluble substance and solvents. The parameter S_VW_ was determined according to Equation (1).

(1) $$S_{VW}=\sqrt{\delta h^{S}}=\sqrt{\frac{\Delta_{\mathrm{soln}}H_{}^{C_{n}H_{2n+2}/\;S}}{V_{X}^{C_{n}H_{2n+2}}}}$$

where *δh^S^* is the parameter of the solvent *S* and is related with specific relative enthalpy of cavity formation, $\Delta_{\mathrm{soln}}H_{}^{C_{n}H_{2n+2}/\;S}$ is the enthalpy of dissolution of any linear alkane in solvent S, and $V_{X}^{C_{n}H_{2n+2}}$ is the McGowan volume.

**Figure 4:**
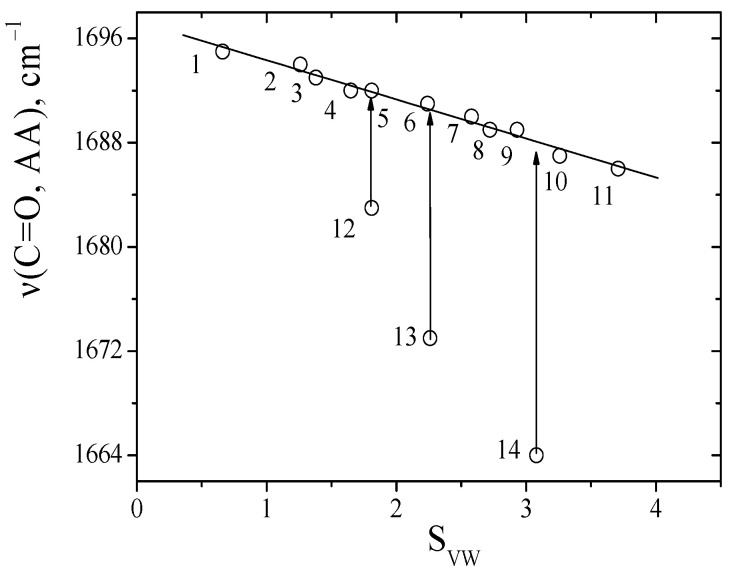
A comparison absorption frequencies of the C=O groups between alanine anhydride and the S_VW_ parameter, values correspond to the data in Table 1.

This parameter and approach to study the contributions of intermolecular interactions of soluble substances in solvents has been successfully tested in a series of works [16,26,27,28,29]. The absorption frequencies of the C=O groups of dipeptides in solvents and the *S_VW_* parameter were compared in this work (Figure 4).

Linear relationships between the *S_VW_* parameter and the absorption frequencies of the C=O groups of anhydride were obtained for inert and aprotic solvents using Equation (2). This made it possible to predict the absorption frequencies of the C=O groups of dipeptides in any solvents.

(2) $$\nu_{s}^{C=O/S}=a_{b}\times S_{VW}+b_{b}$$

Parameters *a* and *b* are correlation parameters; they reflect the sensitivity of the C=O group of dipeptide to nonspecific solvent interactions.

It should be noted that one type of solvation effect was realized for these dipeptides. The parameter accurately describes the solvation effects of the absorption frequencies of the C=O groups of dipeptides (Table S1). At the same time, the solvent sensitivity (parameter *a*) for sarcosine anhydride is lower than it is for alanine anhydride. Similar results were observed for linear amides in the transition from formamide to its substituted analogues [16]. The deviation from experimental frequencies of C=O dipeptide groups in proton donors solvents indicates the solidity of C=O∙∙∙H-O hydrogen bonds (∆ν_HB_) (Table 2). 

This frequency shift varies depending on the choice of the proton donor molecules, which determines their properties accordingly. The smallest shifts in the absorption frequencies of C=O groups of anhydrides are characterized for complexes with chloroform. The shift in the absorption frequencies during the hydrogen bond formation of complexes of C=O groups of alanine and sarcosine anhydride with chloroform is comparable with the values of amides NMFA (9 cm^−1^) [16] and DMFA (11 cm^−1^) [16]. This result shows that the proton acceptor properties of the studied amides as models for cyclic dipeptides are suitable.

The frequency shift values of the C=O groups of alanine and sarcosine anhydride are highest in the medium of water (Table 2). It should be noted that the frequency shift for complexes of dipeptides with water is equal to a shift with methanol that is twice as large. This is related to the ability of water to form an additional hydrogen bond with dipeptides due to the second hydrogen atom in the water molecule. 

### 2.2. Cooperativity Effects of Anhydrides in Proton Donor Solvents

We have previously noted that amides are characterized by the cooperative effects of hydrogen bonds in the medium of associated solvents [14,16]. The cooperative effects of dipeptides with associated molecules were analyzed in this work.

**Figure 5:**
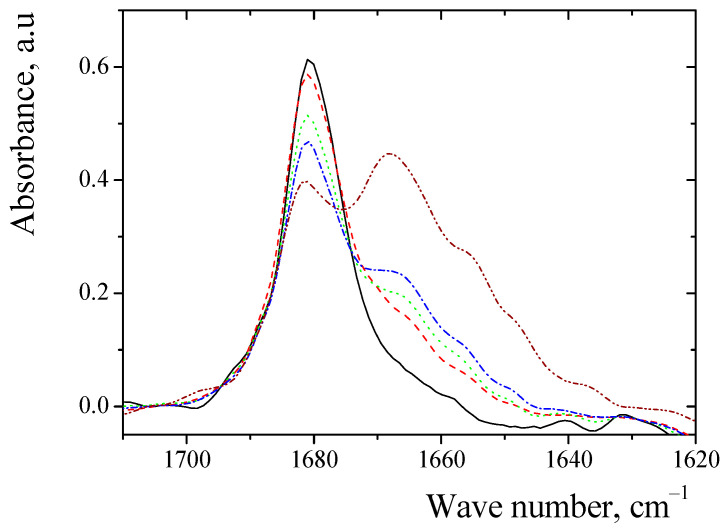
Absorption frequencies of C=O group of complexes between sarcosine anhydride and methanol in benzene: solid line (black)—free anhydride (0.5% wt); dash line (red)—complexes of anhydride (0.5% wt.) with methanol (1% wt.); dot line (green)—complexes of anhydride (0.5% wt.) with methanol (1.5% wt.); dash–dot line (blue)—complexes of anhydride (0.5% wt.) with methanol (2% wt.); dash–dot–dot line (purple)—complexes of anhydride (0.5% wt.) with methanol (10% wt.).

To accurately estimate the cooperative effects of hydrogen bonds (CEHB) of alanine and sarcosine anhydride, we suggested determining the absorption frequencies of C=O groups complexes between anhydride and aliphatic alcohols in proton acceptors solvents. The absorption frequencies of the C=O groups of the complexes of sarcosine anhydride with methanol in benzene are shown in Figure 5.

**Figure 6:**
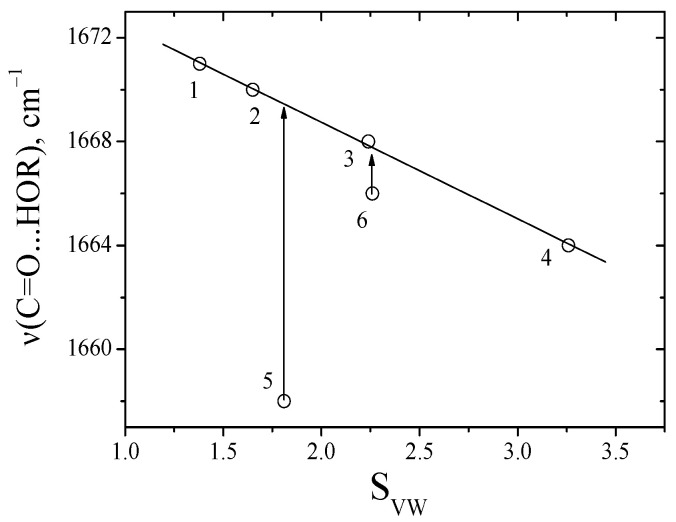
Comparison of the absorption frequencies of the C=O group of the between complex sarcosine anhydride with methanol in solvents and S_VW_, values correspond to the data in Table 2.

In the medium, for benzene, we observed the absorption of the free C=O group of sarcosine anhydride (Figure 5). Increasing the concentration of alcohol gave rise to an additional line, which corresponds to the C=O groups of complex of the anhydrides with alcohol. The absorption frequencies of C=O groups in complexes between anhydrides and aliphatic alcohols in aprotic solvents are given in Table 3. The absorption frequencies of complexes C=O group of dipeptide with alcohols were compared with S_VW_ parameters (Figure 6). In all cases, observed linear correlations with the parameters are presented in Table S2.

The absorption frequencies of C=O groups of alanine and sarcosine anhydride bonding with methanol in media proton donors solvents are independent (Figure 6). It is due to the formation of multi-particle complexes of dipeptides with aliphatic alcohols. Using this difference in the absorption frequencies of C=O groups of diketopiperazines, we determined the CEHB in multi-particle complexes (C=O∙∙∙ (H-OR)_n_), (Equation (3)), Table 4.

(3) $$\Delta \nu_{\mathrm{coop}}^{C=O\ldots {\left(H-OR\right)}_{n}}=\nu_{\exp}^{C=O\ldots {\left(H-OR\right)}_{n}}-\nu_{\mathrm{calc}}^{C=O\ldots H-OR,(1:1)}\;$$

We used Equation (4) to determine the cooperativity coefficients *(A_b_)* of hydrogen bonding in complicated complexes of dipeptides with methanol in proton donor solvent [16,24,26,29].

(4) $$A_{b}=\frac{\nu_{\mathrm{calc}}^{C=O/HOR}-\nu_{\exp}^{C=O\ldots HOR/HOR}}{\nu_{\mathrm{calc}}^{C=O/HOR}-\nu_{\mathrm{calc}}^{C=O\ldots HOR/HOR}}$$

The coefficients of cooperativity of hydrogen bonds of cyclic dipeptides in complexes with alcohols in environment proton donors are listed in Table 4.

It should be noted that the hydrogen bond strengthening in the chloroform medium is significantly higher than it is in alcohol, which is associated with the ability of alcohols to form structures with better compositions, which can weaken the strength of the hydrogen bonds. It was found that the strengthening of hydrogen bonds in multi-particle complexes (C=O∙∙∙ H-OR∙∙∙H-OR), as opposed to equimolar complexes for dipeptides, was from 10 to 16%, which significantly differs from amide complexes, which are characterized by the strengthening of hydrogen bonds from 56 to 133% [16]. These differences can be explained by the conformational flexibility of linear amides, which allows the formation of more complicated structures in multi-particle complexes. Investigated dipeptide systems in multi-particle complexes present considerable interest, since it is the cooperative effects of these objects that predetermine the stability of peptides and proteins.

## 3. Materials and Methods

### 3.1. Materials

Alanine anhydride (AA, 3,6-dimethyl-2,5-piperazinedione) and sarcosine anhydride (SA, 1,4-dimethyl-2,5-piperazinedione) are commercial products with at least 99% purity, and they were dried before the experiments. The organic compounds were purchased from Aldrich (purity >98%). The compounds studied as solvents were dried and purified according to the method in [30]. The water content was estimated by Karl Fischer’s titration method; the content did not exceed 0.05%. Water was prepared by double distillation followed by purification with the system Thermo Scientific Easy Pure II (USA); the electrical resistivity of water was at least Ω = 18.2 ΩΩm.

### 3.2. Method

IR was measured using a Bruker Vector-22 FT-IR spectrometer (Billerica, MA, USA). All absorption spectra were measured in the range from 400 to 4000 cm^−1^. The interferograms were recorded with a resolution of 1 cm^−1^ and Fourier transformed using a Blackman–Harris apodization function. The number of scans used in a particular experiment was 64. We used KBr (0.266 and 0.545 mm) and CaF2 (0.020, 0.545 or 1 mm) cells. At the overlap the bands of C=O groups of cyclic dipeptide (in practically for alanine anhydride), the band was divided into components using Peak Fit 4.0 software.

## 4. Conclusions

In this work, the interaction between cyclic anhydrides in aprotic and proton donor solvents was analyzed. We studied the absorption frequencies of C=O groups of alanine and sarcosine anhydride both in the free state and in complexes with bases by infrared spectroscopy. The influence of intermolecular interactions on the absorption frequencies of C=O groups of anhydrides in solution was discussed. It was shown that the shifts of the C=O groups of dipeptides in the chloroform medium are comparable to those of linear amides. In multi-particle complexes of dipeptides with aliphatic alcohols, the hydrogen bond enhancement was from 10 to 16%, which is significantly less than it is for amide complexes. The paper discusses the influence of the structural specific features of cyclic dipeptides on their intermolecular interactions in solutions. The prospects of this research lie in the possibility of the comparative analysis of cyclic dipeptides with their linear analogues. The practical significance of the evaluation is the influence of the structure and structure of dipeptides on the strength of the complexes formed.

## Figures and Tables

**Table 1:** Absorption frequency of the C=O stretching vibrations of alanine anhydride (AA) and sarcosine anhydride (SA) in solvents (cm^−1^) with parameter S_VW_ (kJ^1/2^∙cm^−3/2^∙10^−2^).

№	Solvent	S_VW_^1^	AA	SA
1	Triethylamine	0.66	1695	1686
2	Diethyl ether	1.26	1694	1683
3	Carbon tetrachloride	1.38	1693	1682
4	Toluene	1.63	1692	1682
5	Tetrahydrofurane	1.81	1692	1681
6	Benzene	2.24	1691	1680
7	Pyridine	2.58	1690	1677
8	Propionitrile	2.72	1689	1677
9	1,2-Dichloroethane	2.93	1689	1676
10	Acetonitrile	3.26	1687	1674
11	Nitromethane	3.71	1686	1673
12	Chloroform	1.86	1683	1671
13	Methanol	2.26	1673	1667
14	Water	3.08	1664	1652

^1^ Data were taken from [16].

**Table 2:** Calculated absorption frequency of C=O group of anhydrides in proton donor solvents.

Solvent (S)	Alanine Anhydride	Sarcosine Anhydride
Chloroform	1692 (9)	1681 (10)
Methanol	1691 (17)	1679 (13)
Water	1688 (25)	1675 (23)

**Table 3:** Absorption frequency of C=O group of alanine (AA) and sarcosine (SA) anhydrides in complexes with methanol in different solvents (cm^−1^) with parameter S_VW_ (kJ^1/2^∙cm^−3/2^∙10^−2^).

№	Solvent (S)	S_VW_	Alanine Anhydride	Sarcosine Anhydride
1	Carbon tetrachloride	1.38	1677	1671
2	Toluene	1.65	1676	1670
3	Benzene	2.24	1675	1668
4	Acetonitrile	3.26	1672	1664
5	Chloroform	1.81	1665	1658
6	Methanol	2.26	1673	1666

**Table 4:** The parameters A_b_ of alanine (AA) and sarcosine (SA) anhydride with methanol in the media chloroform and alcohols.

Proton Donor (RXH)	AA	SA
Chloroform	1.67 (10.8)	1.99 (11.5)
Methanol	1.10 (1.7)	1.16 (1.8)

## Data Availability

The data presented in this study are available in the article.

## Supplementary Materials

The following supporting information can be downloaded at: https://www.mdpi.com/article/10.3390/ijms24076724/s1.
