# Supplementary material for: The Solvation Effect of C=O Group of Cyclic Anhydrides in Solution
Source: Int J Mol Sci. 2023 Apr 4;24(7):6724. doi: 10.3390/ijms24076724 (PMC10095546; doi:10.3390/ijms24076724)
Supplement: Supplementary file 1 [file ijms-24-06724-s001.zip › ijms-2269089-supplementary.pdf]

SUPPLEMENTARY MATERIAL

The solvation effect of C=O group of cyclic anhydrides in solution

Ilnaz T. Rakipov*, Artem A. Petrov, Aydar A. Akhmadiyarov, Artashes A. Khachatrian, Boris. N. Solomonov

Department of Physical Chemistry, Kazan Federal University, Kremlevskaya 18, Kazan, 420008, Russia

[1]To whom correspondence concerning the solution calorimetry experiments and hydrogen bond analysis should be addressed, E-mail**:** ilnaz0805@gmail.com

Table S1. The correlation parameters between the frequencies of the C=O groups of alanine and sarcosine anhydride in solvents and the parameter S_{VW} .

Anhydrides	*a*	*b*	R	S_D	N
Alanine anhydride	-3.00	1697.3	0.991	0.40	11
Sarcosine anhydride	-4.32	1688.7	0.992	0.54	11

Table S2. Parameters of correlation (R, correlation coefficient; S_0, standard deviation) between the frequencies of the C=O groups in the complexes of alanine and sarcosine anhydride with methanol in aprotic solvents and the parameter S_{VW} .

Anhydrides	*a*	*b*	R	S_D	N
Alanine anhydride	-2.58	1680.5	0.995	0.27	4
Sarcosine anhydride	-3.71	1676.2	0.999	0.12	4
